# The Sensitivity and Specificity of Novel Primers for Detection of *BRAF*^*V600E*^ Mutation

**DOI:** 10.31557/APJCP.2020.21.11.3191

**Published:** 2020-11

**Authors:** Bizhar A Tayeb, Howard J Pringle

**Affiliations:** 1 *Molecular Pathology and Toxicology, Department of Molecular Microbiology, Central Laboratory in Ibrahim Al-khalil Zakho, Duhok, Kurdistan Region of Iraq. *; 2 *Department of Cancer Studies and Molecular Medicine, University of Leicester, Leicester, UK.*

**Keywords:** BRAF^V600E^, early detection, low abundance, primer sensitivity, QUASA, qPCR

## Abstract

**Objective:**

This study aimed to evaluate the sensitivity and specificity of a PCR-based novel technique for the detection of *BRAF* mutation in early stages of the cancer.

**Methods:**

Different lengths of primer sets, ranging from 8 bp to 20 bp, were designed and used in this study. These primers were developed by applying on cancer cell lines. After that, the sensitivity and specificity of the methodology was evaluated by making serial dilutions.

**Results:**

The quantitative allele specific discrimination PCR (QUASAqPCR) primer with 14 bp length was sensitive enough to detect significantly 1:1,000 ratio of *BRAF*^V600E^ to wild-type background (P = 0.011), when using 150 nanograms of DNA from cell lines in the reactions.

**Conclusion:**

High sensitivity and specificity levels of QUASA-qPCR method can improve diagnostic accuracy for BRAF mutation testing in patients at early stages of cancers and help stratify the appropriate choice of treatment.

## Introduction

Cancer is a serious problem facing the world, especially in countries with a westernised lifestyle (Ma et al., 2006). The number of new cancer cases diagnosed every year is about 10 – 12 million worldwide (WHO, 2010). Cancers occur due to some changes in the cell behaviour, which can be caused by genetic alterations and accumulations of mutations, especially in tumour suppressor genes (TSGs) and oncogenes (King et al., 2006). Although the majority of the cancer cases were reported in the less developed countries in the past, nowadays it has become also a health challenge in more developed countries (Ma et al., 2006). This issue needs to be looked at carefully and measures need to be taken to overcome this major health problem.

Epidermal growth factor (EGF) and receptor tyrosine kinases (RTK) family play an important role in the transformation of a signal from an extracellular growth factor to the cell, regulating gene expression, and triggering cell proliferation (Carpenter et al., 1990). The EGF receptor (EGFR; also known as ErbB1 or HER1) is the first discovered tyrosine kinase receptor. Three additional family members have also been identified, namely ErbB2 (HER2), ErbB3 (HER3), and ErbB4 (HER4) (Ciardiello et al., 2001; McKinnell et al., 2006). To express gene transcription inside the nucleus by a signal from a growth factor, a series of events is required. For example, binding of a growth factor to its receptor, receptor dimerization, autophosphorylation, activation of intracellular transducers (RAS) and a cascade of serine/threonine kinases, and regulation of gene expression transcription factors (Normanno et al., 2006; Pecorino, 2012). 

The Mitogen-activated protein kinase (MAPK) cascade is initiated on the cell surface by RTK and is considered as a linear pathway for a number of molecules (Normanno et al., 2006). It stimulates gene transcription in the nucleus by extracellular signal-regulated kinase (ERK) (Friday and Adjei, 2006). The pathway calls for many intermediate proteins, such as the RAS family. This family includes v-Ki-ras2 Kirsten rat sarcoma viral oncogene homolog (KRAS), RAF family, v-raf murine sarcoma viral oncogenes homolog B1 (BRAF), and the MEK. Since RAF and RAS oncogenes are members of this pathway, they play a prominent role in the biological downstream biomarkers of the EGFR (Armaghany et al., 2012). Therefore, KRAS and BRAF mutations are key drivers in a variety of tumorigenesis, leading to inappropriate functioning of the majority of cellular responses (Lawrence et al., 2008). Once KRAS is activated, it contributes to the activation of the serine/threonine kinase RAF (McKinnell et al., 2006). 

Three forms of RAF have been identified in mammals, namely CRAF (RAF-1), ARAF, and BRAF. Among these forms, only BRAF is directly activated once it binds to the activated RAS, while the other two forms require further modifications (Wan et al., 2004). Once its kinase function is activated through interaction with RAS, RAF then interacts with a family of proteins known as mitogen-activated protein kinase kinases (MAPKK1 and 2 or MEK1 and 2). All the three MAPK pathways increase the activity of the kinase about one thousand-fold (McKinnell et al., 2006) and act as a general potential mechanism that involves multiple signalling pathways, resulting in different cellular responses (Pecorino, 2012). The sporadic form of cancers is caused by the activation of proto-oncogenes like BRAF and KRAS as well as inactivation of some tumour suppressor genes (TSGs), such as APC and TP53 (17p13.1), or MMR genes like MLH1 and MSH2 (Pancione et al., 2012; Rasuck et al., 2012). 

**Figure 1:**
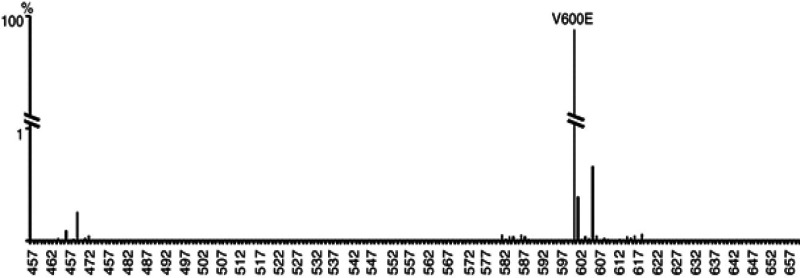
Somatic Mutations of the *BRAF *Gene Recognised in Human Tumour Samples. The V600E missense mutation makes up more than 90% of the somatic mutation defined in human tumour biological samples

More than 40 diverse mutations have been determined within the BRAF gene in human most cancers (Chakraborty et al., 2012; De et al., 2012). It has been proved that BRAF is mutated in numerous malignancies, including 50 to 70% of malignant melanomas, about 45% of papillary thyroid cancers, and 10 to 17% of colorectal malignancies. In addition, it has been recognized in ovarian, breast, and lung cancers (Cantwell-Dorris et al., 2011; Davies et al., 2002). The vast majority (90%) of BRAF mutations are represented by a thymine to adenine single-base change at position 1,799 (Figure 1). This particular missense mutation, located in exon 15, results in a big change at codon 600 which substitutes glutamate for valine (V600E) (Cantwell-Dorris et al., 2011; Wan et al., 2004). This phosphomimetic change renders *BRAF*^V600E^ in a constitutively active and lively state (Garnett et al., 2004), and subsequently activating the RAS/RAF/MEK/ERK cascade leading to abnormal cellular behaviour (De et al., 2012). It is believed that the *BRAF*^V600E^ is frequently associated with an early event of most carcinogenic precursor lesions or occur sporadically (Magnin et al., 2011). 

Given that most cancers are preventable and curable as well as amendable to treatment in early events, the researchers are trying to find approaches to improve the diagnostic tools, treatment strategies, and prevention measures by detecting the cancer in its early stages (Arends et al., 2013). The aims of the current study were to validate a new mode of allele specific discrimination quantitative PCR (QUASAqPCR) and to improve the sensitivity and specificity of new tools targeting the *BRAF* oncogene by using modified primers and PCR cycling conditions. 

## Materials and Methods

*Cell lines and culture*

Three human cell lines, namelyHCT-116, HT-29, and A375-P were obtained from the American Type Culture Collection (ATCC) to do this study. HCT-116 is a large intestine colon carcinoma cell line and a *BRAF*^V600E^ wild type (WT) cancer cell line. HT-29 is a Caucasian colon carcinoma and a *BRAF*^V600E^ heterozygous cell line. These two cell lines were grown in the culture medium McCoy’s 5a + 2mM Glutamine + 10% foetal calf serum (FCS). A375-P is a malignant melanoma and a homozygous *BRAF*^V600E^ mutant cell line. The culture medium for this cell line is Roswell Park Memorial Institute (RPMI) with L-Glutamine and 10% FCS. Those cell lines are widely studied and authenticated. More information about them can be found on www.sanger.ac.uk and www.hpacultures.org.uk. 

*DNA extraction from cell lines*

DNA of these three cell lines (i.e. HCT-116, HT-29, and A375-P) was extracted using QIAamp DNA mini kit (Qiagen, Hilden, Germany). According to the manufacture instructions, 20 microliters (µl) of protease was placed into an eppendorf tube, 200µl of cell pellet was added to the same tube, and the mixture was vortexed for 15 seconds. Then, 200µl of lysis buffer (AL) was added to the mixture and vortexed well, and the mixture was incubated in a water-bath at 56°C for 10 minutes. After a short centrifugation,, 200µl of absolute ethanol (at room temperature) was added and again vortexed followed by a brief spin. The aliquots were transferred to a spin column and centrifuged at 8,000 rpm for one minute. The spin column was put into a new collection tube, and 500µl buffer AW1 was added to denature proteins in the samples. Next, y a second centrifugation was done at 8000 rpm for one minute. The spin tube was again transferred to a new collection tube, and 500µl buffer AW2 was applied to remove non-specific bindings to the column. The mixture was span at 14,000 rpm for 3 minutes following one extra minute at the same speed after the collection tube was emptied. Finally, 200µl of elution buffer (AE) was added to the spin column and incubated at room temperature for 5 minutes followed by spinning down the mixture at 8000 rpm for one minute. Concentration of DNA was quantified by using Nanodrop Spectrophotometer (ND-100 Technologies, V3.2.1, USA), and the isolated DNAs were stored at 4°C. 

*QUASA primer-probe design*

**Figure 2:**
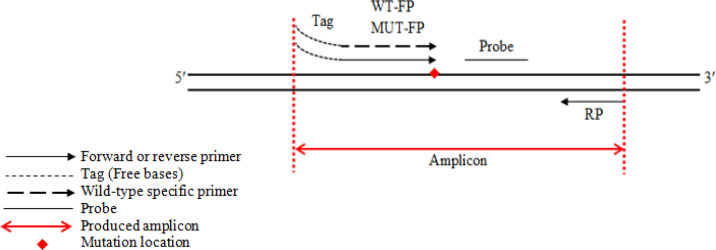
QUASA Primer Designs Used for the *BRAF*^V600E^ Mutation Analysis for the Real-Time PCR. The above Figure indicates the positions of the primers and probe used for the *BRAF*^V600E^ real-time PCR. Forward mutation-unspecific primer (dotted arrows) and forward mutation-specific primer share the same probe. The mutation location (•) occurs at the codon 600. Tiny dots indicate the tag at the 5ʹ -end forward primers for QUASAqPCR. Abbreviations: FP, forward primer; RP, reverse primer; WT, wild-type specific forward primer; MUT, mutant-specific primer. Tag indicates some free independent bases at the 5ʹ of each forward primer. Italic A at the 3ʹ of mutant primer illustrates the mismatch base at codon 600 of the *BRAF* gene

 The QUASA primers require a free sequence (tag) at the 5ʹ-end terminal of the gene sequence. The ‘tag’ is a short independent sequence consisting of some bases of oligonucleotides attached to the 5ʹ -end of both forward primers (Figure 2). Few forward primers, starting from 8, 10, 12, 14, 16, 18, and 20 nucleotides, were designed for this study (Table 1). Since the tag is included in the produced amplicons, the size of the amplicons from primers 8, 10, and 12 nucleotides are 72-bp, while the 14-mer primers give an amplicon of 69-bp. The 16-base wild-type specific primer gives an amplicon of 69 bp; whereas, the same primer but mutation-specific gives a 71 bp amplicon because each of them has a different ‘tag’ at the 5ʹ-end. Finally, both the 18 and 20 nucleotide primers give an amplicon of 73 base pair. The forward primers, in different lengths and tags, are both mutation-specific and mutant non-specific primers for amplification of mutant allele and wild-type allele respectively. One reverse primer (5ʹ-ATCCAGACAACTGTTCAAACTGATG-3ʹ) and one probe (5ʹ-TCTCGATGGAGTGGGTC-3ʹ) were used to amplify both mutant and WT alleles. All these oligonucleotides were designed using the primer express3.0 software (Applied Biosystems, Cheshire, UK), and the oligonucleotides were checked for the melting temperature (Tm) by using the Primer 3 software. In addition, all sequences were checked for self-molecular or intermolecular annealing with the same software. Furthermore, the sequences were performed local alignment analyses by the BLAST program (http://blast.ncbi.nlm.nih.gov/Blast.cgi) to confirm the specificity of the designed primers. The designed oligonucleotides were synthesised and purified by Sigma (Sigma-Aldrich Company, Dorset, UK), giving different amplicon sizes because different primers were used in different lengths. 

*Standard dilution of c.1799T>A (V600E) mutated DNA into WT DNA*

*BRAF* ^V600E^ WT DNA was extracted from HCT-116, and mutant *BRAF*^V600E^ DNA was extracted form A375-P cell lines. These two types of DNA were mixed and serially diluted as 50%, 25%, 12.5%, 6.25%, 3.12%, 1.5%, 0.78%, 0.4%, 0.2%, 0.1%, 0.05%, 0.02%, and 0.01% mutant into WT DNA background to assess and check the sensitivity of the QUASAqPCR method and to compare the sensitivity levels among those primers used in the current study. For serial dilutions, the reactions were initially performed from about 150 nanogram (ng) / 3µl of DNA from cell line controls. 

*Real-time PCR*

Quantitative real-time polymerase chain reaction (qPCR) was done with a final volume of 10 µl of reaction in each well. QUASAqPCR reactions were performed using 5 µl of TaqMan Genotyping Master Mix (Applied Biosystems, catalog No. 4371355, Cheshire, UK,), 0.6 µl of 10 pmol of each forward and reverse primers, 0.2 µl of TaqMan probe at 1:50 dilution, and 0.6 µl of H2O with 3 µl DNA (a final volume of 10 µl in each well). Mastermixes were prepared as instructed by the manufacturer and distributed in a 96-well plate. The reactions in QUASAqPCR method starting from 10 ng/ 3µl for cell line DNA, 150 ng/ 3µl for cell line DNA in serial dilutions. PCR amplicons were amplified in the standard mode running (~2 h) on a StepOne plus machine (Applied Biosystems) and based on thermocycling conditions defined in Table 2. Amplifications were completed at least in duplicate to ensure the reproducibility of the assay. Real-time data and threshold cycle (Ct), sometimes called quantification cycle (Cq), values were collected during the last 50 cycles of the amplification using StepOne Software v2.2.2 (Applied Biosystems). Samples with delta (∆) Ct less than reference ∆Ct were considered positive for the mutation of *BRAF*^V600E^, and ∆Ct = Ct of mutant primer – Ct of WT primer (Richter et al., 2013). 

*Statistical analysis*

Statistical analysis was carried out to evaluate the sensitivity analysis of the QUASA-qPCR on cell lines’ DNAs at different concentration points. For data analysis, the GraphPad Prism version 6 (GraphPad software, California, United States) and Microsoft Excel (2010) software were used and one-way analysis of variance (ANOVA) and Tukey’s test were run. Results with p value < 0.05 were considered as statistically significant.

## Results

*QUASAqPCR primers specificity and selectivity*

**Figure 3:**
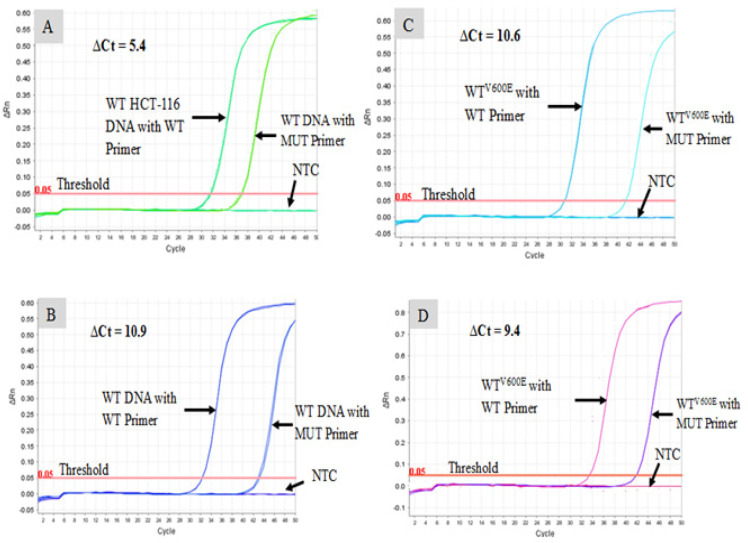
Specificity of QUASAqPCR Primers. Amplification plots of *BRAF*^V600E^ fluorescence versus cycle number show the specificity of QUASAqPCR primers. The Figures demonstrate the ∆Ct of the WT DNA (HCT-116) amplifying with both WT and mutant primers (at least duplicate samples). The result shows that ∆Ct values (Ct mutant primer - Ct WT primer) in these particular plots are (A) 5.4 cycles for the primer in 12-base; (B)10.9 for 14-base primer; (C) 10.6 in case of 16-base primer; (D) 9.4 for the primer with 18 bases length. Abbreviations: WT, wild-type; MUT, mutant; NTC, no template (water) control

The WT DNA was quantified by the WT-specific primers at lower threshold cycle (Ct), and the same DNA was quantified with mutation-specific primers at higher Ct. The specificity and selectivity of QUASA-qPCR assays were determined by obtaining the difference in the Ct values, known as the ‘delta Ct’, where using a delta (∆) Ct method (Ct [mutant primer] - Ct [WT primer]). The ∆Ct values were varied depending on several factors, including the length of the primers. For example, the ∆Ct values of QUASAqPCR primers composed of 12, 14, 16, and 18 nucleotides were 5.4, 10.9, 10.6, and 9.4, respectively (Figure 3). In addition, no PCR products were obtained in the negative (no template) control with the template of this method, assuming that the primers were specific to *BRAF*^V600E^.

*Analytical sensitivity of the QUASAqPCR method*

**Figure 4:**
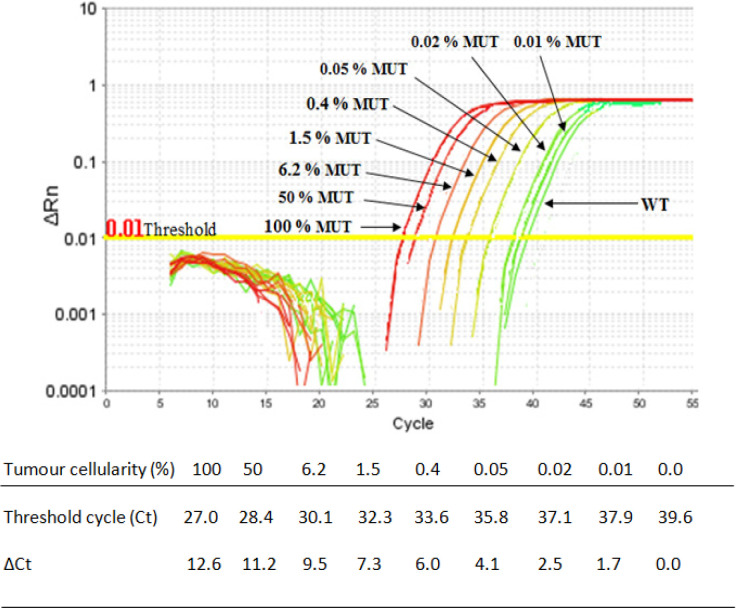
Analytical Sensitivity of QUASAqPCR. Upper panel: Mutant *BRAF*^V600E^ DNA was extracted from A375-P cell line and WT DNA was extracted from HCT-116 cell line and serially diluted. Amplification plots obtained for wild type (WT) DNA and samples containing known percentage, 100, 50, 6.2, 1.5, 0.4, 0.05, 0.02, and 0.01% (single samples) *BRAF*^V600E^ mutated alleles (MUT) to WT background and amplifying with mutant primer (14-base). Lower panel: The Ct and ∆Ct values of QUASAqPCR, where the ∆Ct = Ct in mutant primer – Ct of WT DNA (0.0%). A very low amount of the BRAFV600E mutations was detectable in the reaction

Analytical sensitivity of the QUASAqPCR method was tested by serially diluting DNA from the *BRAF*^V600E^ mutated A375-P cell line into WT DNA from HCT-116 cell line. Duplicates of WT DNA (0% mutant) were included in this experiment as a reference, as well as negative (water) control. There was no amplification of water in the reactions. All concentrations of the mutant DNA were used to be quantified w ith the mutant primer at Cts between 27 and 38. The higher concentrations of the mutant DNA were quantified at the lower Ct values and vice versa. The WT DNA started to react non-specifically with the mutant primer after the Ct 40. The ∆Cts of the 100%, 50%, 6.2%, 1.5%, 0.4%, 0.05%, 0.02%, and 0.01% mutant were 12.6, 11.2, 9.5, 7.3, 6, 4.1, 2.5, and 1.7, respectively (Figure 4). This might suggest that the QUASAqPCR method can detect a very low amount of the *BRAF*^V600E^ mutation present in WT background in the reactions.

*qPCR repeatability and reproducibility*

Repeatability (i.e. short-term precision or intra-assay variance) refers to the precision of results in the same run, confirming the robustness of the assay. Repeatability was measured in this study by determining the coefficient of variation (CV) from Ct values of duplicated samples from serial dilutions (50%, 25%, 12.5%, 6.25%, 3.12%, 1.5%, 0.78%, 0.4%, 0.2%, 0.1%, 0.05%, 0.02%, and 0.01% ) of mutant DNA A375-P into WT DNA from HCT-116 cell lines run in the same plate. The QUASAqPCR coefficient of variation for *BRAF*^V600E^ ranged between 0.14% and 4.37%. Reproducibility (i.e. long-term precision or inter-assay variance) was similarly assessed by calculating the Ct coefficient of variance of the same serial dilutions of mutated DNA mentioned above but run in different days . The CV of the *BRAF*^V600E^ for QUASAqPCR ranged between 0.15% and 2.95%. The results from intra- and inter- assay reproducibility within 10% were considered as satisfactory values (Murray and Lawrence, 1993).

*Limit of detection*

Limit of detection (LOD) is the minimum amount of target DNA sequence that can be detected in a sample with a given level of confidence. For the cell lines, serial dilutions were made for this purpose. QUASAqPCR could detect as low ratio as 0.0005 mutant DNA in a sample in the reaction. This corresponded to 48.8 and 97.6 mutated cells (~ 1% cells) detectable in the reactions, assuming that the *BRAF*^V600E^ mutation was more likely to be heterogeneous.

## Discussion

Activation of the *BRAF* oncogene in all types of malignancies accounts for 10 to 17% (Davies et al., 2002). There are more than 40 different types of *BRAF* mutations occuring in cancers. However, approximately 90% of these mutations are seen at position c.1799T>A of the *BRAF* gene. As a result, an amino acid is substituted at codon 600 in *BRAF*, from a valine (V) to a glutamic acid (E) (V600E) (Davies et al., 2002). A group of monoclonal antibodies with or without chemotherapy have been developed to target EGFR in patients with metastatic cancers (Behl et al., 2012). However, patients with the *BRAF*^V600E^ mutation tend to acquire a lack of response to the monoclonal antibodies targeted EGFR (Mao et al., 2011). Since *BRAF* is a crucial effector downstream of RAS in the MAP kinase and it is the driver oncogene, it might be a potential marker for targeting KRAS mutated cases. In addition, the *BRAF* and *KRAS* genes are mutated at a similar phase of tumorigenesis, but they are mutually exclusive (Rajagopalan et al., 2002). Moreover, a retrospective study has shown that ERK signalling in tumours with the *BRAF*^V600E^ is inhibited in the cells treated with RAF inhibitors (Poulikakos et al., 2011). Additionally, a different study has proven that cell lines harbouring *BRAF*^V600E^ mutation are enhanced and more sensitive than the *KRAS* mutations when treated with MAPK/ERK kinase inhibitors (Solit et al., 2006). Therefore, several *BRAF* inhibitors have been used to treat the cases harbouring the *BRAF*^V600E^ mutations (Tie et al., 2011). These are the reasons that the EGFR cascade and particularly *BRAF* oncogene has become of interest in various research searching for novel cancer treatments.

In addition, the American Society of Clinical Oncology (ASCO) and the National Comprehensive Cancer Network (NCCN) suggest that the *BRAF* gene to be evaluated prior making treatment decisions for patients with cancers (Allegra et al., 2009; Anderson, 2001). This might be due to considerably high presence of the *V600E* in the* BRAF* gene and response of *WT BRAF* to some kind of treatments like panitumumab or cetuximab (Di Nicolantonio et al., 2008; Santini et al., 2010). The sensitivity of some existing methods is relatively low and their detection depends on the percentage of mutated cells in the samples ranging between 5 and 20% (Lewandowska et al., 2013). Those methods are unable to detect low percentage mutation, especially in the somatic mutations, because the somatic mutations tend to have a very low abundant mutation like a “needle in a haystack”.

QUASA is an innovative form of qPCR based on allele specific discrimination. Its primers are made in ways that the 3ʹ terminal base overlies the mismatch base. Therefore, the WT primers consult 100% specificity the non-mutated sequence but have an only mismatch base with the mutated sequence (the talk is true of this mutant specific primers). This can be common regarding allele specific PCR along with the actual basic principle which the individual mismatch base will certainly avoid the WT primers effectively prime on the mutated sequence. Nonetheless, this particular basic principle on its own cannot be often enough in order to confer specificity and false positive amplification can be frequent. Therefore, QUASA primers were even more improved to be able to increase the degree of specificity. To start with, the actual primers were designed to use a lower melting Tm in a way that a single base was going to be denatured easily. QUASA primers additionally had a series of independent ‘tag’ in the 5ʹ end. This tag was included in the produced amplicon through the initial round of PCR and it was therefore contained in the particular amplicon for followed cycles. Which means that the particular tagged primers will certainly prime perfectly with this template and therefore drive amplification from the appropriate sequence within following cycels. The QUASA standard protocol worked on a two-stage-cycling tactic. The very first five cycles of PCR worked with a very low annealing temperate of 53ºC. This enabled the lower Tm primers to prime effectively though achieving maximum levels of specificity. Following the first five cycles, the actual annealing temperate was changed to 60ºC (Table 2). Hence, allele specific priming would be successfully blocked and priming happened at the spot that the tagged primers were already integrated. This also pushed extremely effective amplification and also probed cleavage and therefore gained level of responsiveness from the procedure. The QUASA technique needs absolutely no additional primers, clamping primers, modified bases, or even blocking probes to offer the remarkable level of sensitivity with the system and methodology.

Based on QUASA principles mentioned above, few forward primers in different lengths, ranging from 10, 12, 14, 16, 18, and 20 bases oligonucleotides with different tags , were designed to check the sensitivity and specificity of the assay (Table 1). Regarding QUASA method, the 8bp and 10bp oligomer primers had extremely low sensitivity, so they were excluded from the study. However, the ∆Ct values from primers of 12, 14, 16, and 18 bases nucleotides were 5.4, 10.9, 10.6, and 9.4 cycles, respectively (Figure 3). It is well known that the higher ∆Ct primers have hypersensitivity feature as it will allow detecting proportion of mutant DNA alleles among total DNA alleles (Filion, 2012; Mouliere et al., 2013). Clearly, the 14 and 16 base primers are proven to have the highest levels of sensitivity for the detection of the *BRAF*^V600E^, with 0.05% mutant alleles. Not surprisingly, the shorter as well as the longer primers loss their sensitivity. 

A number of previous studies have drawn attention to the fact that the amplicon length might affect the actual sensitivity regarding genotyping as the short amplicon lengths have provided superior solution involving genotypes and also enhanced the level of sensitivity of mutation recognition (Liew et al., 2004; Pichler et al., 2009). However, the amplicon lengths from different primers were similar in the present study. Nevertheless, they were different only in ‘tag’ that attached to the 5ʹ end terminal of each forward primer. For example, the 10-base primers carry a longer ‘tag’ of 15 bases nucloetide, while the 14 and 16-base primers take a short ‘tag’ composed of 8 bases. This could be the reason of differences between the sensitivity levels among the primers.

Consequently, QUASA primers were shown to work more effectively and achieve high specificity and sensitivity for the detection of the *BRAF*^V600E^ mutation in this study. Mutant alleles were significantly detectable down to 1:1,000 mutant into WT DNA ratio for QUASAqPCR . An interesting piece of previous research compared five different methods for the detection of *BRAF*^V600E^ mutation in cutaneous melanoma. In the aforementioned study, ∆Ct of TaqMan allele-specific PCR used to determine the mutation status was less than nine and mutation detection was as low as 2.5% mutant allele (Lade-Keller et al., 2013). That study used a different method called CADMA and it was concluded that this method had more sensitive and was capable to detect 0.078% mutant alleles.

In conclusion, QUASA-qPCR method could significantly detect a very low mutation allele into WT background in reactions. This method for detection of the *BRAF*^V600E^ was extremely accurate, very easily performance and interpreted, and probably reduce the amount of time that clinicians and pathologists as well as patients that wait for the results, may possibly prove to be more affordable tools, and can be done within hours. In addition, this method is not restricted to detection of merely *BRAF* mutations. Further studies are needed in order to validate the findings of this study 

**Figure 5:**
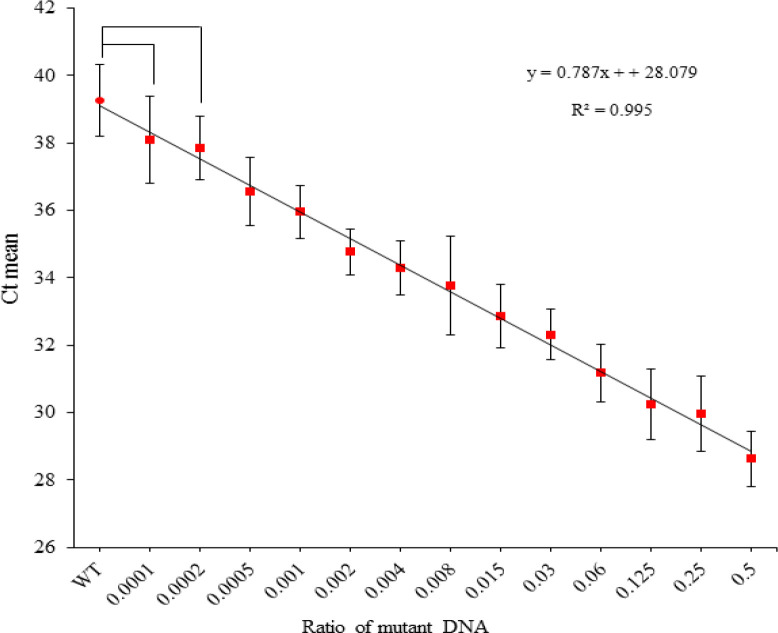
Standard Curve Titration of the QUASAqPCR. Serial dilution of *BRAF*^V600E^ mutated A375-P cell line DNA in wild type (WT), HCT-116 DNA. Red squares correspond to 0.5, 0.25, 0.125, 0.06, 0.03, 0.015, 0.008, 0.004, 0.002, 0.001, 0.0005, 0.0002, 0.0001 ratios of mutant DNA into wild type versus the Ct mean of three runs in different days. The red circle corresponds to the WT reference DNA. The data is represented mean ± SD by One-way ANOVA. Stars like symbols (*) indicate that the values were significantly different from the control (WT) 1* = p < 0.05; 2* = p <0.01; 3* = p < 0.001; 4* = p < 0.0001; ns: non significant (i.e. p value > 0.05) according to the Tukey’s multiple comparisons test in ANOVA

**Table 1:** QUASAqPCR-Modified Primers and Probe. The table shows few forward primers in different lengths and different tags for QUASAqPCR method. A reverse primer and a TaqMan probe were used for the detection of the BRAFV6000E

L	WT/MUT	Tag	Gene sequence	Amplicon size
8	MUT	CAAGTGGCGGCTAGACT	GCTACAGA	72 bp
10	MUT	CAAGTGGCGGCTAGA	TAGCTACAGA	72 bp
12	MUT/WT	CAAGTGGCGGCTA	TCTAGCTACAGT/A	72 bp
14	MUT/WT	GTATGCGC	GGTCTAGCTACAGT/A	69 bp
16	WT/MUT	GCATGCGGCGATCAG	TTGGTCTAGCTACAGA	69-71 bp
18	MUT	CCGTACTC	TTTTGGTCTAGCTACAGT/A	73 bp
20	WT/MUT	GACTCG	GATTTTGGTCTAGCTACAGT/A	73 bp
25	Reverse P	No tag	ATCCAGACAACTGTTCAAACTGATG	
17	Probe	---------	TCTCGATGGAGTGGGTC	

L, length of the primer; WT, wild-type specific forward primers; MUT, mutation-specific primer; Tag, represents some independent bases at the 5ʹ of forward primers in QUASAqPCR method; P, primer; bp, base pair. Italic A at the 3ʹ of mutant primers illustrates the mismatch base at codon 600 of the *BRAF* gene.

**Table 2:** PCR Conditions and Cycling for the QUASAqPCR

Temperature	Time	Cycles	Stage
95°C	10 min	1	Holding stage
95°C	15 s	5	Denaturation (Stage 1)
53°C	20 s	5	Annealing (Stage 1)
95°C	15 s	45	Denaturation (Stage 2)
60°C*	1 min	45	Annealing (Stage 2)

*, Plate reading step; min, minutes; s, seconds; QUASAqPCR, Quantitative allele specific amplification qPCR method.

## References

[B1] Allegra CJ, Jessup JM, Somerfield MR (2009). American Society of Clinical Oncology Provisional Clinical Opinion: Testing for KRAS gene mutations in patients with metastatic colorectal carcinoma to predict response to anti-epidermal growth factor receptor monoclonal antibody therapy. J Clin Oncol.

[B2] Anderson SM (2011). Laboratory methods for KRAS mutation analysis. Exp Rev Mol Diagn.

[B3] Arcila M, Lau C, Nafa K, Ladanyi M 2011) Detection of KRAS and BRAF mutations in colorectal carcinoma roles for high-sensitivity locked nucleic acid-PCR sequencing and broad-spectrum mass spectrometry genotyping. J Mol Diagn.

[B4] Arends MJ (2013). Pathways of colorectal carcinogenesis. Appl Immunohisto M M.

[B5] Armaghany T, Wilson JD, Chu Q, Mills G (2012). Genetic alterations in colorectal cancer. J Gastrointest Cancer.

[B6] Behl AS, Goddard KA, Flottemesch TJ (2012). Cost-effectiveness analysis of screening for KRAS and BRAF mutations in metastatic colorectal cancer. J Natl Cancer Inst.

[B7] Cantwell-Dorris ER, O’Leary JJ, Sheils OM (2011). BRAFV600E: implications for carcinogenesis and molecular therapy. Mol Cancer Ther.

[B8] Carpenter G, Cohen S (1990). Epidermal Growth Factor. J Bio Chemis.

[B9] Chakraborty A, Narkar A, Rajan MGR (2012). BRAFV600E mutation in papillary thyroid carcinoma: Significant association with node metastases and extra thyroidal invasion. Endocr Pathol.

[B10] Ciardiello F, Tortora G (2001). A novel approach in the treatment of cancer: Targeting the Epidermal Growth Factor Receptor. Clin Cancer Res.

[B11] Davies H, Bignell GR, Cox C (2002). Mutations of the BRAF gene in human cancer. Nature.

[B12] De LA, Maiello MR, D’Alessio A, Pergameno M, Normanno N (2012). The RAS/RAF/MEK/ERK and the PI3K/AKT signalling pathways: role in cancer pathogenesis and implications for therapeutic approaches. Expert Opin Ther Pat.

[B13] Di Nicolantonio F, Martini M, Molinari F (2008). Wild-type BRAF is required for response to panitumumab or cetuximab in metastatic colorectal cancer. J Clin Oncol.

[B14] Filion M (2012). Quantitative real-time PCR in applied microbiology.

[B15] Friday BB, Adjei AA (2008). Advances in targeting the Ras/Raf/MEK/Erk mitogen activated protein kinase cascade with MEK inhibitors for cancer therapy. Clin Cancer Res.

[B16] Gabriel J (2007). The Biology of Cancer.

[B17] Garnett MJ, Marais R (2004). Guilty as charged: B-RAF is a human oncogene. Cancer Cell.

[B18] Harding MC, Sloan CD, Merrill RM (2018). Transitions from heart disease to cancer as the leading cause of death in US states, 1999–2016. Prev Chronic Dis.

[B20] Lade-Keller J, Rømer KM, Guldberg P (2013). Evaluation of BRAF mutation testing methodologies in formalin-fixed, paraffin-embedded cutaneous melanomas. J Mol Diagn.

[B21] Lawrence MC, Jivan A, Shao C (2008). The roles of MAPKs in disease. Cell Res.

[B22] Lewandowska MA, Joawicki W, Zurawski B (2013). KRAS and BRAF mutation analysis in colorectal adenocarcinoma specimens with a low percentage of tumor cells. Mol Diagn Ther.

[B23] Liew M, Pryor R, Palais R (2004). Genotyping of single-nucleotide polymorphisms by high-resolution melting of small amplicons. Clin Chem.

[B24] Ma X, Yu H (2006). Global burden of cancer. Yale J Biol Med.

[B25] Magnin S, Viel E, Baraquin A (2011). A multiplex SNaPshot assay as a rapid method for detecting KRAS and BRAF mutations in advanced colorectal cancers. J Mol Diagn.

[B26] Mao C, Liao RY, Qiu LX (2011). BRAF V600E mutation and resistance to anti-EGFR monoclonal antibodies in patients with metastatic colorectal cancer: a meta-analysis. Mol Bio Rep.

[B27] McKinnell RG, Parchment RE, Perantoni AO, Damjanov I, Pierce GB (2006). The Biological Basis of Cancer.

[B28] Mouliere F, El MS, Gongora C (2013). Circulating cell-free DNA from colorectal cancer patients may reveal high KRAS or BRAF mutation load. Transl Oncol.

[B29] Murray A, Lawrence GP (1993). How should the repeatability of clinical measurements be analysed? An assessment of analysis techniques with data from cardiovascular autonomic function tests. Q J Med.

[B30] Normanno N, Luca AD, Bianco C (2006). Epidermal growth factor receptor (EGFR) signaling in cancer. Gene.

[B32] Pecorino L (2012). Molecular Biology of Cancer.

[B33] Pichler M, Balic M, Stadelmeyer E (2009). Evaluation of high-resolution melting analysis as a diagnostic tool to detect the BRAF V600E mutation in colorectal tumors. J Mol Diagn.

[B34] Poulikakos PI, Persaud Y, Janakiraman M (2011). RAF inhibitor resistance is mediated by dimerization of aberrantly spliced BRAF (V600E). Nature.

[B35] Prahallad A, Sun C, Huang S (2012). Unresponsiveness of colon cancer to BRAF(V600E) inhibition through feedback activation of EGFR. Nature.

[B36] Rajagopalan H, Bardelli A, Lengauer C (2002). Tumorigenesis: RAF/RAS oncogenes and mismatch-repair status. Nature.

[B37] Rasuck CG, Leite SMO, Komatsuzaki F (2012). Association between methylation in mismatch repair genes, V600E BRAF mutation and microsatellite instability in colorectal cancer patients. Mol Biol Rep.

[B38] Richter A, Grieu F, Carrello A (2013). A multisite blinded study for the detection of BRAF mutations in formalin-fixed, paraffin-embedded malignant melanoma. Sci Rep.

[B39] Santini D, Spoto C, Loupakis F (2010). High concordance of BRAF status between primary colorectal tumours and related metastatic sites: implications for clinical practice. Ann Oncol.

[B40] Solit DB, Garraway LA, Pratilas CA (2006). BRAF mutation predicts sensitivity to MEK inhibition. Nature.

[B41] Tie J, Gibbs P, Lipton L (2011). Optimizing targeted therapeutic development: analysis of a colorectal cancer patient population with the BRAFV600E mutation. Int J Cancer.

[B42] Wan PTC, Garnett MJ, Roe SM (2004). Mechanism of Activation of the RAF-ERK Signaling Pathway by Oncogenic Mutations of B-RAF. Cell.

[B43] World Health Organisation (WHO) (2010). Global status report on noncommunicable diseases 2010.

